# Psychosocial Factors Associated with Substance Abuse and Anxiety on Immigrant and U.S. Born Latinos

**DOI:** 10.13188/2330-2178.1000028

**Published:** 2016-06-17

**Authors:** Roberto Lopez-Tamayo, Julia DiGangi, Gloria Segovia, Gabriela Leon, Josefina Alvarez, Leonard A. Jason

**Affiliations:** 1 Center for Community Research, DePaul University, Chicago, USA; 2 Adler School of Professional Psychology, Adler University, Chicago, USA

**Keywords:** Psychosocial factors, Substance abuse, Anxiety, Latino immigrants, U.S. born Latinos, Multi group path analysis, Relapse prevention

## Abstract

Latinos are exposed to adverse psychosocial factors that impact their health outcomes. Given the heterogeneity and rapid growth of this population, there is an urgent need to understand the mechanisms through which psychosocial factors impact substance abuse and anxiety between immigrant and U.S. born Latino adults. The present study employs a multi-group path analysis using Mplus 7.2 to examine generational differences in the paths between affiliation culture, years of formal education, contact with important people, and length of full-time employment to substance abuse and anxiety in immigrant and U.S. born Latino adults who completed substance abuse treatment. A total of 131 participants (Mage= 36.3, SD ± 10.5, 86.3% males, 48.1% non-U.S. born with a mean length of stay of 19 years in the U.S. (SD ± 13.71) in recovery from substance abuse completed self-report measures. Results from the multi-group path analysis suggest that being more affiliated to the U.S. culture is associated with substance abuse, whereas years of formal education and longer full-time employment is associated with reduced anxiety in the immigrant group. Conversely, frequent contact with important people and affiliation to the U.S. culture are associated with fewer years of substance abuse, whereas longer full-time employment is associated with substance abuse in the U.S. born group. Anxiety and substance abuse was correlated only in the U.S. born group. The implications of these findings are discussed.

## Psychosocial Factors Associated with Substance Abuse on Immigrant and U.S. Born Latinos

With a population exceeding 51 million, Latinos are the largest and fastest growing minority in the United States [1]. Despite the growth rate experienced by Latinos - three times faster than the total U.S. population (14%), there is limited research on substance abuse treatment (SAT) completion among Latinos [2,3]. National data revealed that 9.7% of Latinos met criteria for substance abuse and dependence in 2010 [4]. However, from 2003 to 2011, Latinos were less likely than European Americans to have received substance abuse treatment (SAT) (9% vs. 10.5% respectively). Among those in need of services, 7.7% received treatment and only 58% completed treatment or were transferred to a control environment [4]. Although aggregate rates of substance use for Latinos are lower than national averages, exposure to unique social and contextual factors is likely to increase the disparities in SAT completion among Latino subgroups [4–6].

The sparse research on access and substance abuse treatment utilization among Latinos shows mixed results [7–9]. Although a few studies indicate that Latinos access SAT at the same rate than European Americans and African Americans, most studies found that Latinos have poorer SAT outcomes than their European American counterparts [10–14]. Specifically, Latinos encounter more barriers to access SAT, receive fewer services, are less satisfied with treatment, report higher likelihood of unmet need, and are more likely to drop out of SAT than European Americans [6,9,13,15–21]. Taken together, the above literature illustrates the need for research to understand key individual and contextual factors that inform substance use prevention and services for Latinos [9,22,23].

## Comorbidity between Acculturation and Anxiety

The co-occurrence between substance abuse and anxiety on Latinos who complete SAT deserves consideration. A review of the literature shows that anxiety disorders were present in nearly 75% of individuals with substance use disorders (SUDs), partially supporting the self-medication pathway [24,25]. The long-term use of alcohol and drugs impacts neurotransmitter systems implicated in the pathophysiology of anxiety-related disorders (i.e. corticostriatal-limbic motivational, mesolimbic dopamine, glutamate, and gamma-amino-butyric acid [GABA] pathways), unmasking symptoms that may contribute to substance use relapse [26,27]. For individuals in substance use recovery, anxiety from substance use withdrawal and environmental triggers may increase the likelihood for relapse [28]. Recently, a study using national data found differences in anxiety and substance abuse rates among Latinos based on generational status [29]. Given that pathways to substance use and anxiety may differ among Latinos, there is the need for substance use models that examine the impact of psychosocial factors on specific groups [30].

## Psychosocial Factors in Relation to Substance Abuse and Anxiety

### Generational status and psychosocial factors

Currently, Latino immigrants comprise 36% of the total Latino population and most of them are middle age (age 35 and over) [1,14]. Although nearly 40% of Latinos are born in the U.S., Immigrants comprise a larger segment of the Latino population, with a significant number of foreign-born Latinos arriving after 1990 [31,32]. Besides differences in age of arrival and length of time living in the U.S., acculturation is key to understanding the social and contextual factors that may lead to relapse on both, non-U.S. born and U.S. born Latino recovery addicts [33]. Acculturation is defined as the “dual process of cultural and psychological change that takes place as a result of contact between two or more cultural groups and their individual members” [34]. Research on community samples of Latinos indicates that immigrants are more likely to have low acculturation with the U.S. culture and low English proficiency; low educational attainment; fewer occupational opportunities; and reduced social networks due to family separation [35–38]. The cumulative impact of the aforementioned adverse factors has been linked to neighborhood disadvantage exposure, increased environmental stressors, high-risk conditions, and limited social support, which, in turn, increase the likelihood of using alcohol and illicit substances as coping mechanisms [39–43]. Conversely, U.S. born Latinos have higher substance abuse rates and psychiatric disorders, are likely to be more affiliated with the U.S. culture, speak English, endorse nontraditional family values than their immigrant counterparts [35,44–47].

Substance abuse literature has documented existing disparities in substance abuse within Latinos based on level of acculturation, country of origin, years of residence in the U.S., and generational status [23,48]. Studies using nonclinical samples of Latinos suggest that the longer immigrants live in the U.S., the more they resemble their U.S. born counterparts in substance abuse rates [35,49,50]. However, little is known about the psychosocial factors that hinder the recovery process of Latino adults who have completed SAT [33]. On one of the few studies comparing European American and Latinos receiving SAT found that Latinos were likely to be court-referred, from low socioeconomic status (SES), had higher drug than alcohol problem, and high level of criminal activity [51]. A study using national data to examine ethnic and gender differences among individuals seeking SAT found differences in substance use rates, not only based on gender and ethnicity, but also between those seeking services and those found in community samples [22]. Given the heterogeneity (i.e., generational status, acculturation) and dynamic growth of this population, there is the need for research that shed light on the factors that increase the likelihood of relapse in immigrants and U.S. born Latinos who have completed SAT [15,22,48].

### Affiliation to the U.S. culture

The acquisition of U.S. cultural norms related to acculturation has been linked to mental health problems and substance abuse among Latinos [28,35,52]. However, sources of stress may vary between immigrants and U.S. born Latinos. Latino immigrants contend with the challenges associated with adapting to the mainstream society [53]. The stress that stems from adjusting to new social norms, learning a new language, and experiencing discrimination is referred to in the literature as acculturative stress [54,55]. Some unique stressors include, difficulties integrating to the mainstream culture and providing economical support to family back in their country of origin may lead to increased substance use [40]. Thus, substance use rates among Latino immigrants may increase as a function of the stress experienced from adapting to the U.S. mainstream culture and exposure to disadvantaged environments [56,57]. Although their U.S. born counterparts may not experience significant stress from the acculturation process, stress from navigating between two cultures (e.g. home culture and mainstream culture) and perceived discrimination and marginalization may be important sources of anxiety for this group [48,58]. Overall, use of alcohol and drugs is seemed as a coping strategy to reduce stress from social inequalities and the acculturation process [59–62].

### Education and occupational opportunities

Fewer years of formal education and poor employment history have been found to increase the likelihood of relapse [15,63]. A recent study using national data found that unemployment and precarious housing largely contribute to lower treatment completion among Latinos [6]. Studies using community samples found that few years of formal education impact the chances of securing a full-time job, have work-related benefits, and have the ability to afford better living conditions [64]. Although few years of education and low English proficiency do not prevent Latino immigrants from securing employment, poor working conditions and low wages may put them at risk for increased anxiety [39]. The cumulative impact of low educational attainment has also been linked to neighborhood disadvantage exposure, high-risk conditions and limited social support, increasing the likelihood of using alcohol and illicit substances as a coping mechanism [42,65].

### Support for substance use from important people

The collectivistic orientation of the Latino culture emphasizes the importance of social networks for their well-being [66]. However, when family members and social networks exert a negative influence by supporting the use of alcohol and drugs, the odds of relapse increase exponentially [2,67]. Support for substance use from social networks has been identified as a risk factor to substance use relapse [68,69]. A study on individuals living in communal recovery homes found that social networks that encourage drinking or drug use predicts relapse among those working on their recovery process [70]. In the same vein, studies on treatment ecology have explored individual and environmental factors in relation to alcohol and drug relapse [71]. In a study conducted on 180 individuals who completed SAT, researchers found that two years after treatment completion, participation in leisure activities with substance abusers and need for resources (e.g. employment, housing) contributed to alcohol and drug relapse [72]. These findings indicate the need for research to understand key cultural and social aspects that inform substance use prevention and services for Latinos [7,9].

## The Present Study

**Figure 1:**
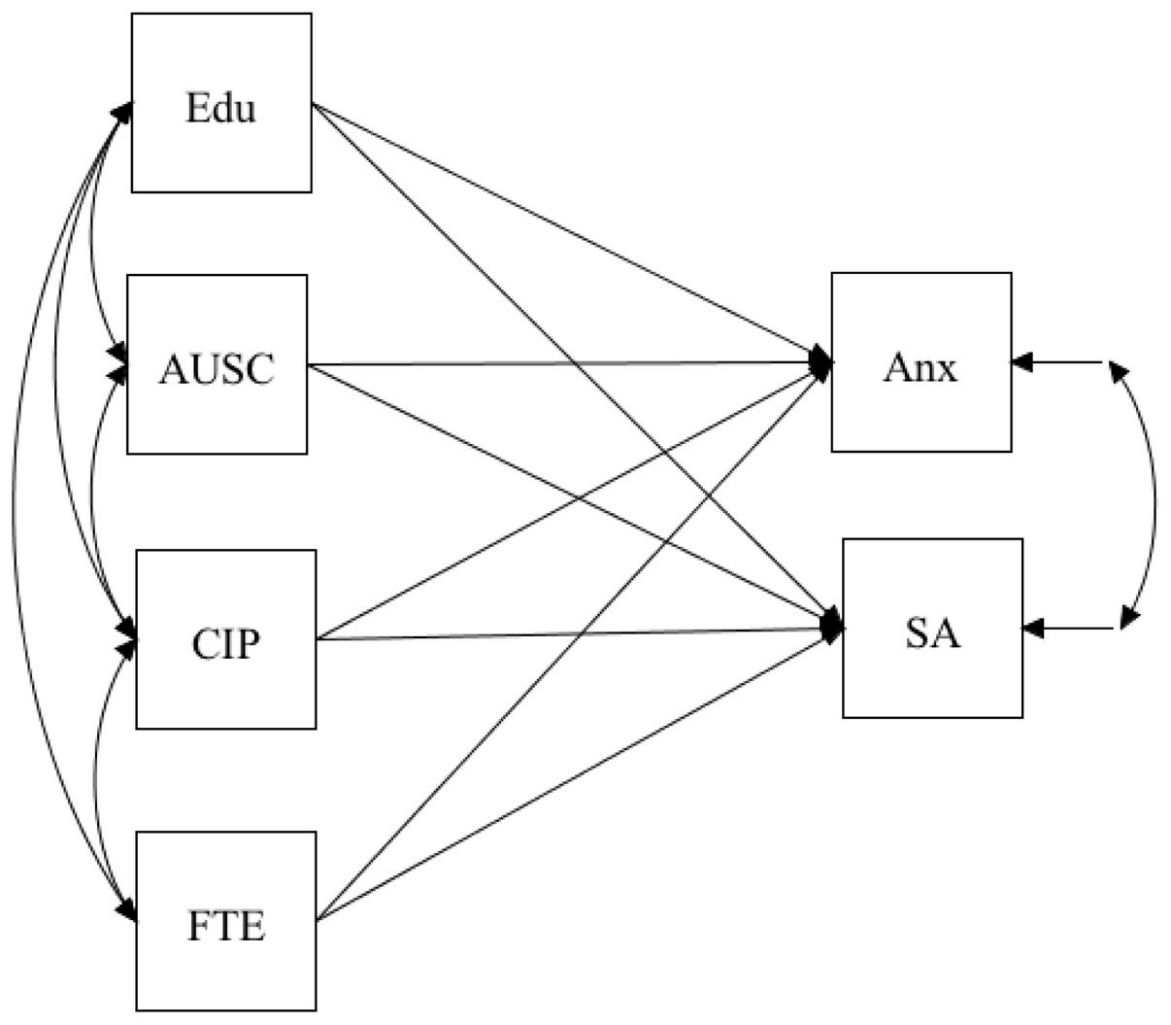
Theoretical model of the proposed associations for the immigrant and U.S. born groups. **Note:** Edu: Years of formal education; AUSC: Affiliation to the U.S. culture; CIP: Contact with important people; FTE: History of full-time employment; Anx: Anxiety; SA: Days using alcohol and drugs in the last six months.

The dearth of research on clinical populations who completed SAT remains significant for Latinos [73]. Research that examines unique psychosocial factors that increase the likelihood for relapse is needed to inform current treatment services and develop relapse prevention programs tailored for this population [21]. The present study proposes the use of a socioecological model of acculturation, with generational status (i.e. non-U.S. born vs. U.S. born) as moderator of the association between psychosocial factors and substance abuse and anxiety (Figure 1) [74]. Specifically, we propose a multi-group model that examines the association between affiliation to the U.S. mainstream culture, years of formal education, full-time employment history and social support, and substance use and anxiety on a sample of immigrants and U.S. born Latino adults who completed SAT. Having a better understanding of the contextual and psychosocial factors that Latinos contend with after completing SAT would help reduce the risk of psychiatric and legal problems, involvement with the criminal justice system, unemployment, and social exclusion among Latinos working on their recovery [22].

## Methods

### Participants

Participants for this study were part of a larger NIH-funded study that examined community-based recovery homes for Latinos in recovery from substance abuse [68]. A total of 131 Latinos were recruited from multiple substance abuse treatment programs and health facilities from a large metropolitan area in the Midwest. The criteria for participation were 1) being of Latino background, and 2) either having completed substance abuse treatment or having lived in a controlled environment. Of the 131 participants (M_age_ = 36.3; SD ± 10.5), 113 were males (86.3%) and 18 females (13.7%). Nearly half of the participants immigrated from Mexico, Puerto Rico, and other Central American countries (48.1%), with a mean length of stay of 19.2 years (SD ± 13.71) in the U.S. The majority of the participants had alcohol and substance abuse treatment previously (n = 124), while for seven participants it was their first time in treatment. For sociodemographic characteristics see Table 1.

### Setting and procedures

Recruitment of participants took place from fall 2009 to spring 2012 for a larger NIH-funded study aimed to examine recovery homes for Latinos in recovery from substance abuse [68,75]. A cadre of bilingual/bicultural Oxford House alumni and research assistants was formed to facilitate outreach, recruitment and assessment of Latino participants. Research assistants utilized internet search engines (i.e. Google, Yahoo) and statewide databases of health services and mental health providers to generate a list of substance and treatment programs, hospitals, and community-based agencies servicing Latinos. The outreach strategy consisted of contacting these sites via phone and email to introduce the study. A team of Oxford House alumni, two of them Latinos, worked to establish ties with staff and potential participants at various treatment centers. Recruiters provided information on community-based recovery home options, described the nature of the study to potential participants, and facilitated the interview process. All participants were given an explanation about the nature, purpose and goals of the study before signing consent forms. Participants were interviewed in their language of preference (i.e. English or Spanish). Interviews took place at treatment facilities, a private location within an Oxford House, or at the DePaul Center for Community Research.

After completing the interview, participants received $30 as compensation for their participation.

### Measures

#### Demographics

A 24-item demographic questionnaire was used to collect participants’ age, gender, place of birth, country of origin, and treatment setting.

#### Country of origin

Participants were asked to report their place of birth and were assigned either to the immigrant or U.S. born groups. Puerto Ricans who were born on the island were placed in the immigrant group. We acknowledge that Puerto Ricans are U.S. citizens by birth. However, given the fact Puerto Rico endorses traditional cultural norms similar to those of other Latin American countries, we determine to place Puerto Ricans born in the island with other Latino immigrants.

#### Substance abuse

The Addiction Severity Index (ASI), 5^th^ Edition, assesses problems during the individual’s lifetime and during the 30 days prior to the interview in seven areas: alcohol use, drug use, illegal activity, interpersonal and family relations, medical problems, employment, and psychiatric problems [76]. A sample item includes “how many days in the last 30 days have you experienced alcohol problems?” High scores indicate greater problem severity. English and Spanish versions of the ASI were used, depending on participants’ stated language preference. The ASI has been used extensively in research with multicultural samples and has demonstrated strong psychometric properties. The 5^th^ Edition of the ASI has been used with Latino participants in several large studies and yielded valid data [7,22,77]. The Spanish translation was back translated and pilot tested prior to undergoing a validation study [78]. Correlations for the test-retest reliability of the English and Spanish versions of the ASI range from 0.80 to 0.90. For the present study, a substance use index was computed using mean of the alcohol, heroin, cocaine, cannabis, and more than one substance items. Higher scores indicate greater substance use lifetime. In addition, the items years of formal education and full-time employment pattern were used in the analysis.

#### Acculturation

The Bidimensional Acculturation Scale for Hispanics (BAS) is a 24-item, 4-point Likert-type (1 = low or not well to 4 = high or very well) self-report measure of English and Spanish use as a proxy for acculturation [79]. Three subscales measure language use, linguistic proficiency, and use of electronic media subscales in both Spanish and English. An item sample of the language subscale includes “how often do you speak English?” The Hispanic and Non-Hispanic domain scores are derived from the total scale, where scores higher than 2.5 suggest biculturalism. Good to high internal consistency (α = 0.81 – 0.97) and high correlation with other behavioral measures of acculturation, such as generation in the U.S. and proportion of life spent in the U.S. are reported [79]. For the present study the Non-Hispanic subscale was used to assess for affiliation to the U.S. culture.

#### Support for substance use

The Important People and Activities Inventory (IPA) examines the impact of social network by asking participants questions about their relationships with significant people [80]. Participants were asked to list up to 12 people that are important and they had contact within the past 4 months. Participants also reported the four most important people and the most liked among those listed. Indices were derived to obtain the number of people in the network, amount of contact with one’s network, substance use status and frequency with which network members use substances, support for drinking among most important people, and the average support for use among the most important people. The Important People portion of the scale has demonstrated good internal consistency (α = 0.80) [81]. The IPA was translated into Spanish by a bilingual-bicultural team composed of a psychologist and three research assistants, who focused on semantic equivalence. The average contact with important people was used to assess social support for the present study.

#### Anxiety

The Trauma Symptom Checklist 40 (TSC-40) is a 40-item, 4-point Likert-type (0 = *never* to 3 = *often*) measure that evaluates symptomatology in adults associated with traumatic experiences [82]. The symptoms are rated according to frequency of occurrence over the last two months. The measure consists of six subscales: anxiety, depression, dissociation, sexual abuse trauma index (SATI), sexual problems, and sleep disturbance, as well as the total score. A sample item of the anxiety subscale includes “in the last two months, have you feeling tense all of the time?”, studies using the TSC-40 report good internal consistency for the full scale ( = 0.89 and 0.91) and for the subscales (α = 0.66 to 0.77). The TSC-40 was translated into Spanish by three bilingual-bicultural research assistants, who focused on semantic equivalence. The anxiety subscale was used to assess anxiety level in participants.

## Results

Preliminary analyses, using pairwise deletion to address the issue of missing data, were conducted to determine descriptive statistics. The final sample used for the model analysis was 131 participants (n = 63 immigrant, n = 68 U.S. born), with a mean age of 36.15 years. Means, standard deviations and correlations for all study variables are presented in Table 2. Bivariate correlations indicate that, in the immigrant group, years of formal education were negatively correlated with anxiety, being male is positively correlated with anxiety, and age was positively correlated with substance abuse lifetime and full-time employment pattern. Conversely, in the U.S. born group, substance abuse lifetime was negatively correlated with average contact with important people, age was positively correlated with substance use and full-time employment pattern and negatively correlated with affiliation to the U.S. culture (Table 3).

Independent-samples t-tests were conducted to compare the impact of the aforementioned psychosocial factors on immigrant and U.S. born Latinos. Results from the t-tests revealed that U.S. born Latinos were more affiliated to the U.S. Culture, t (129) = 9.18, p < 0.001; completed more years of formal education, t (129) = 3.26, p < 0.001; and had more contact with important people, t (129) = 2.83, p = < 0.01, than their immigrant counterparts. In contrast, there was a significant difference in age between the immigrant (M = 39.14, SD = 10.90) and U.S. born Latino (M = 33.66, SD = 9.39) groups, t (129) = 3.09, p < 0.01. Conversely, there was no significant difference between the immigrant and U.S. born groups in terms of substance abuse, anxiety, and employment pattern.

A multiple-group analysis was conducted to compare immigrant and U.S. born Latinos on the impact of psychosocial factors on substance abuse and anxiety using the Mplus computer software, version 7 [83]. Maximum Likelihood analysis was employed to determine the overall fit of the model to the data. Model fit was evaluated using multiple indicators of fit, including the comparative fit index (CFI), the chi-square statistics, the Tucker Lewis Index (TLI), the root-mean-square residual error of approximation (RMSEA), and the standardized root mean square residual (SRMR) [84].

Results from the model with paths freely estimated indicated displayed poor model fit indices (X^2^ = 6.61, df = 3, p = 0.08, CFI = 0.92, TLI = 0.47, RMSEA = 0.13, RMSEA 90% C.I. = 0.00 – 0.27, SRMR = 0.03). Next, recurrent nonsignificant pathways were constrained to zero (i.e. contact with important people substance abuse; contact with important people anxiety in the immigrant group; full-time employment anxiety in both, the immigrant and U.S. born group) to improve model fit indices. The constrained multiple-group model yielded an adequate fit to the data as indicated by the values on the following fit indices (χ^2^ = 8.12, df = 7, p = 0.32, CFI = 0.98, TLI = 0.93, RMSEA = 0.04 (RMSEA 90% C.I. = 0.01 – 0.16, SRMR = 0.03). Next, a model with all paths constrained to equal was estimated (χ^2^ = 18.72, df = 10, p = 0.04, CFI = 0.82, TLI = 0.61, RMSEA = 0.11, RMSEA 90% C.I. = 0.02 – 0.19, SRMR = 0.07). The model fit for the fully constrained model was significantly worse than both, the model with freely estimated paths and the trimmed model. To test for group difference, the chi-square from the trimmed model was compared to the chi-square from a model with paths constrained to equal. The two models were significantly different (Δχ^2^ = 10.6, Δdf = 3, p = 0.01), suggesting group differences (Table 3).

In the immigrant group, after controlling for age, affiliation to the U.S. culture was significantly positively associated with substance abuse lifetime (β = 0.28, p = 0.007). Similarly, years of formal education (β = −0.39, p = 0.01) and full-time employment pattern (β = 0.38, p = 0.001) were significantly negatively associated with anxiety. In other words, for each 1 standard deviation (SD) increase in affiliation to the U.S. culture, there is a 0.28 SD increase in years of substance use. Conversely, for each 1 standard deviation (SD) increase in years of formal education and length of full-time employment, there is a −0.38 SD and −0.37 SD decrease in anxiety rates. Substance abuse lifetime and anxiety were not related (Table 4 and Figure 1).

**Figure 2:**
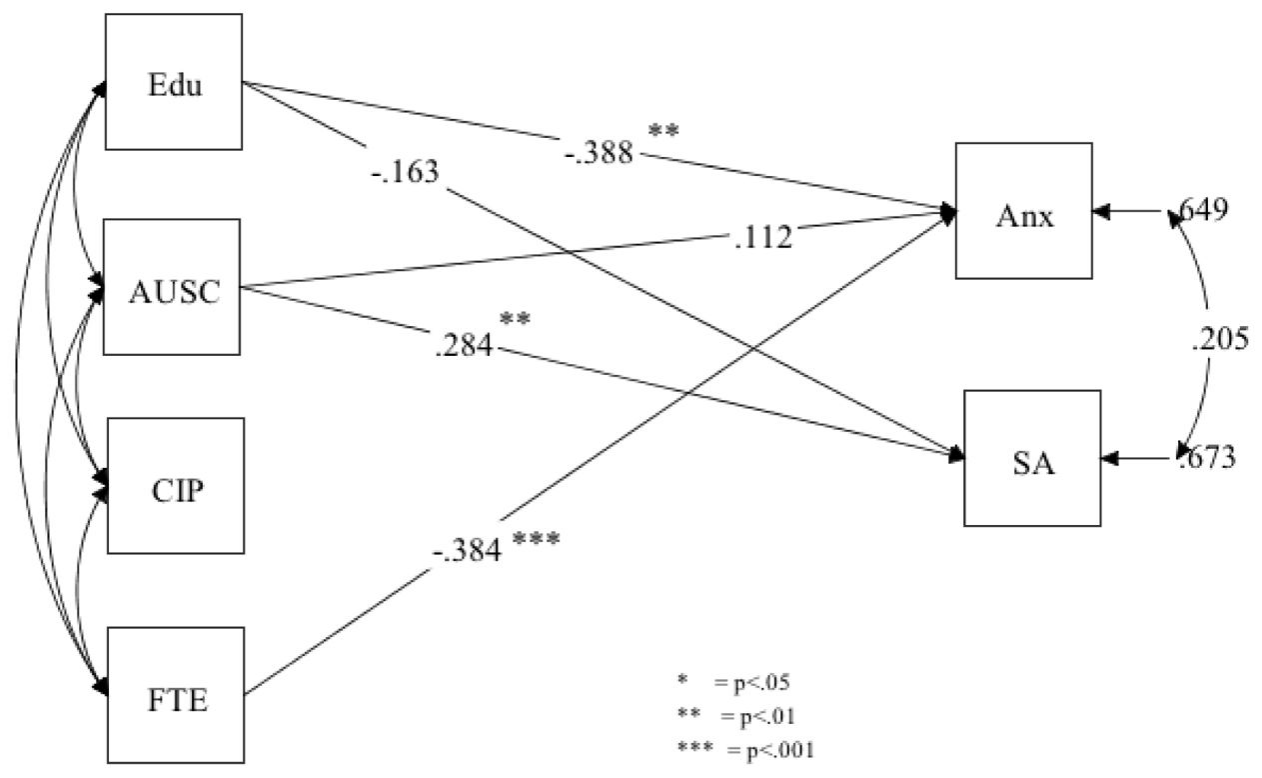
Standardized coefficients for the trimmed model for the immigrant Latino group.

**Figure 3:**
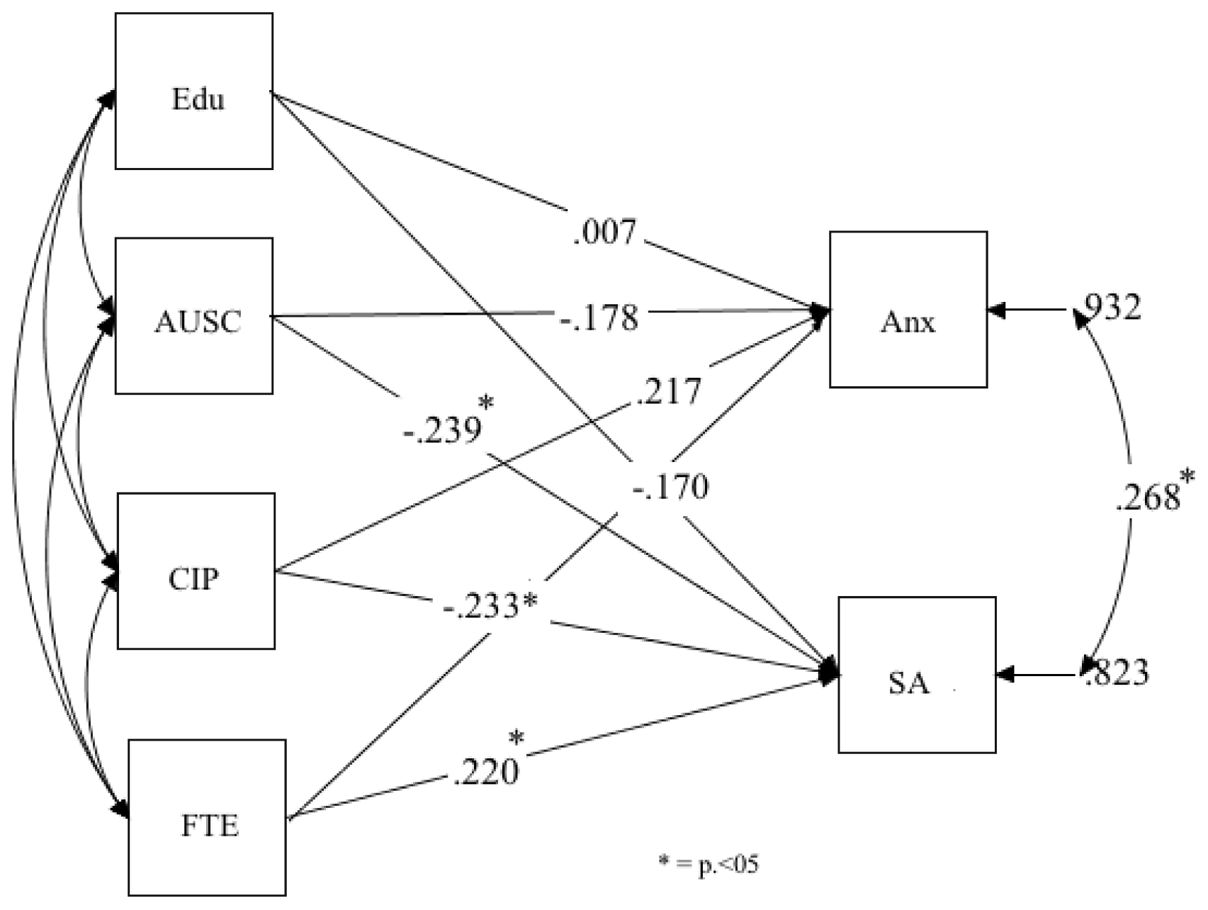
Standardized coefficients for the trimmed model for the U.S. born group.

Conversely, in the U.S. born group, average contact with important people (β = −0.23, p = 0.03), and affiliation to the U.S. culture (β = −0.24, p = 0.02) were negatively associated with substance use lifetime. Conversely, full-time employment pattern was significantly positively associated with substance use lifetime (β = 0.22, p = 0.04). Substance use lifetime and anxiety were significantly positively correlated (r = 0.27, p = 0.02). In other words, for each 1 standard deviation (SD) increase in average contact with important people and affiliation to the U.S. culture, there is a −0.24 SD and a −0.22 decrease in years of substance use. Conversely, for each 1 standard deviation (SD) increase in length of full-time employment, there is a 0.22 SD increase in years of substance abuse (Figures 2 and 3). Overall, results from the immigrant and U.S. born groups support the hypothesis that generational status moderates the association between psychosocial factors and substance abuse and anxiety.

## Discussion

The purpose of the present study was to examine whether generational differences moderate the association between psychosocial factors and anxiety on a sample of Latinos who completed SAT. Results from the multi-group analysis revealed significant differences between the two groups, relative to the outcomes of interest. Specifically, in the immigrant group, after controlling for age, being more affiliated to the U.S. mainstream culture was associated with more years of substance use. Conversely, contact with important people, years of formal education, and length of full-time employment were not related to years of substance use. Results for the U.S. born group indicate that, after controlling for age, longer full-time employment was associated with more years of substance use, whereas contact with important people and affiliation to the U.S. culture were associated with fewer years of substance use. Years of formal education were not related to substance abuse.

With regards to anxiety, in the immigrant group and after controlling for age, more years of formal education and longer full-time employment were associated with reduced anxiety. Conversely, affiliation to the U.S. culture and contact with important people were not related to anxiety. In the U.S. born group, none of the pathways was related to anxiety. Lastly, substance abuse was positively correlated with anxiety in the U.S. born group, but not in the immigrant group.

These findings illustrate substantial differences between the immigrant and U.S. born group in the direction and strength of the paths from psychosocial factors to substance abuse and from psychosocial factors to anxiety. Although some research have focused on disparities during early stages of treatment and outpatient SAT, the present study advances previous research on substance abuse on Latinos by examining generational differences on a sample of Latinos who completed SAT [22,73,85]. Of significance is that substance abuse and anxiety rates among the immigrant group are similar than those of the U.S. born group. These findings are partially consistent with the immigrant paradox literature, which asserts that the longer immigrants live in the U.S., the more they resemble their U.S. born counterparts in anxiety and substance abuse rates [48,86,87]. It is important to note that participants in the immigrant group have a mean length of stay in the U.S. of 19 years, suggesting that have adopted lifestyle patters of the U.S. mainstream culture.

Our results showed that affiliation to the U.S. culture was associated with more years of substance use among immigrants, but it was not related to anxiety. Although Latino immigrants, particularly middle-age adults experience greater difficulty to adopt social norms and values of the U.S. culture, those who live in cultural enclaves, or communities with high concentration of immigrants, may be less exposed to discrimination and receive support from community members [29,58,88]. Another explanation for the nonsignificant association between affiliation to the U.S. culture and anxiety is the coping mechanisms employed by Latino immigrants when experiencing anxiety. Some studies suggest that fatalismo, or resignation for adverse situations, and religion, may reduce anxiety in Latino immigrants [5,60,89]. It is also plausible that the tendency to internalize anxiety mask anxiety-like symptoms on this population [87].

The result indicating that more affiliation with U.S. mainstream culture was associated with fewer years of substance use seems to contradict previous studies conducted on community samples of U.S. born Latinos [35,48]. is unexpected finding may be explained by the use of a substance use index for the present study. Disaggregation of illicit substances and alcohol use may offer a more accurate portrait of this association [5]. An alternative explanation is that U.S. born Latinos may not only endorse affiliation to the U.S. culture, but also endorsement of traditional culture. However, the affiliation to traditional culture was not included in the proposed multi-group model.

Our results suggest that more years of formal education and longer full-time employment reduce anxiety in the immigrant group. These findings shed light on the extent to which the above factors may protect Latino immigrants from anxiety. It is plausible that Latino immigrants with more years of formal education are more likely to learn the English language and U.S. cultural norms, facilitating the acculturation process. In addition, for most Latino immigrants who moved to the U.S. looking for better jobs, being gainfully employed may elicit a sense of success. Given the co-occurrence between anxiety and substance abuse, recovery oriented services should be tailored to the needs of the population. Specifically, securing employment and stable housing have been reported as an important recovery goal among Latinos working in their recovery [68]. In a study conducted on individuals who completed SAT found that those who returned to more stable environments (i.e. stable job and housing) showed better outcomes at 2-year and 10-year follow-up [90]. Overall, lower stress levels and more stable environments contribute to substance use cessation [71,91].

The findings for the U.S. born group indicating that frequent contact with important people was associated with fewer years of substance use is consistent with the social control theory [92]. Strong bonds with family members and friends motivate individuals to engage in prosocial behavior and refrain from engaging in substance use [92]. When social support is weak or absent (e.g. dysfunctional families, friends who promote use of alcohol or drugs), individuals are less prone to adhere to conventional norms and more likely to engage in alcohol and drug misuse [93]. Several studies conducted on individuals who live in communal recovery settings found that social support, particularly from parents and family members play a key role in supporting individuals through their recovery process [33,67,70,94]. Given the importance of family involvement in the recovery process, assessing for specific social support from family members who do not accept or promote substance use is key to promote sobriety [7,67,70].

Lastly, the path from longer full-time employment to years of substance abuse seems to be in function of being affiliated to the U.S. culture and having more years of formal education. More important, the results indicating that anxiety was correlated with substance abuse in the U.S. born group, but not in the immigrant group suggest unique sources of anxiety that affect this group. Support for the link between anxiety and substance abuse can be found in studies conducted on community samples [95,96]. However, more research on the co-occurrence between anxiety and substance abuse in both groups is needed to inform current SAT and substance use relapse prevention programs.

Contrary to our hypothesis, the same path was not correlated in the immigrant group. It is plausible that Latinos may underreport anxiety symptoms due to artifactual level explanations [48]. Several studies indicated that the use of diagnostic assessment instruments may fail to detect intensity of symptom experiences that are common among Latinos, such as “nervios” [97,98]. Extant literature indicates that the presence of psychological distress in the form of somatic symptoms (i.e. headaches, chronic pain, and diminished quality of life) may vary across Latino subcultures, making it more difficult to detect [99,100]. In addition, the stigma associated to mental illness, sense of disempowerment, and a perceived normative experience of the immigration experience (i.e. all immigrants deal with the same issues) may mask anxiety levels among Latino immigrants [14,101].

## Limitations

There were several limitations, including a small sample that did not allow for exploration of other psychosocial factors (i.e. involvement with the criminal justice system). The cross-sectional nature of the present study does not allow for causal inferences. Participants on the present study completed SAT in multiple settings, which may reduce generalizability to similar populations. In addition, in an attempt to test the proposed model on Latinos who grew up in Latin American countries, we included Puerto Ricans who were born in the Island (*n* = 30, 47.6% of the participants assigned to the immigrant group) into this group. Although Puerto Ricans may contend with discrimination, low educational attainment, and have difficulties acculturating to the U.S. mainstream culture, their status as U.S. citizens afford them access to resources that non-U.S. citizens do not have. is may have driven the high affiliation to the U.S. mainstream culture in tandem with the length of residence in the U.S. Further studies are needed to explore the impact of psychosocial factors within Latino immigrants. Another limitation is that the low number of Latino women in the dataset did not allow for an examination of gender differences within each group. Lastly, most participants were from Puerto Rican and Mexican background (in both immigrant and U.S. born groups), which may limit generalization of findings to other Latino subcultures.

Subsequent studies are needed to inform and develop culturally-tailored SAT and substance use prevention programs for Latino subgroups (i.e. by generational status, country of origin, acculturation). More important, to develop effective substance use prevention programs for individuals who completed SAT, research is needed to understand the underlying processes through which psychosocial factors impact clinical samples of Latinos to inform ways to intervene. Specifically, there is the need for more studies to understand the culture of reception wherein Latinos are immersed, how symptoms manifest and the responses to prevention among this ethnic group in order to culturally informed prevention programs [102]. By the same token, there is a need to identify protective factors and community resources that prevent substance use relapse. Given the collectivistic orientation of Latino cultures, Latinos may benefit from community-based programs that promote sobriety. Mounting evidence suggests that communal recovery settings promote a sober and inviting environment for Latinos to continue their recovery [68,75]. Our findings suggest that the practice of screening for substance use and dependence should be complemented with the assessment of anxiety symptoms as well as important psychosocial factors discussed in the present study. Additionally, our results could be used to inform substance abuse counselors on important aspects to address in their clients’ aftercare plan. In sum, a better understanding the extent to which psychosocial factors impact Latinos who completed SAT is key to provide resources and skills needed to continue their recovery process.

## Figures and Tables

**Table 1:** Sociodemographic characteristics by Latino immigrant (n = 63) and U.S. Born Latino (n = 68) groups.

		Latino Immigrants (n = 63) M (SD)	U.S. Born Latinos (Mainland) (n = 68) M (SD)
Age		39.1 (10.9)	33.7 (9.4)
Education		10.4 (2.9)	11.8 (1.8)
		**% (n)**	
Sex	Male	96.8 (61)	76.5 (52)
	Female	3.2 (2)	23.5 (16)
Marital Status	Married	6.3 (4)	3.0 (2)
	Separated	22.2 (14)	13.4 (9)
	Divorced	22.2 (14)	19.4 (13)
	Never married	49.2 (31)	64.2 (43)
Country of Origin	U. S. born (mainland)	-	100 (67)
	Puerto Rico^1^	47.6 (30)	-
	Mexico	41.3 (26)	-
	Cuba	4.8 (3)	-
	El Salvador	3.2 (2)	-
	Guatemala	3.2 (2)	-
Employment Pattern^2^	Full-time	49.2 (30)	36.9 (24)
	Part-time	31.1 (19)	32.3 (21)
	Unemployment	19.7 (12)	30.8 (20)
Substance of Major Problem	Alcohol	23.8 (15)	16.4 (11)
	Heroin/Opiates/Analgesics	15.9 (10)	26.9 (18)
	Cocaine	12.7 (8)	9.0 (6)
	Cannabis/Amphetamines	7.9 (5)	11.9 (8)
	Alcohol & one or more drugs	31.7 (20)	31.3 (21)
	More than one, not alcohol	7.9 (5)	3.0 (2)
Prior Substance Abuse Treatment	No	6.3 (4)	4.5 (3)
	Yes	93.7 (59)	95.5 (64)
History of Incarceration	No	25.4 (16)	19.4 (13)
	Yes	74.6 (47)	80.6 (54)
Legal Status (on Parole/Probation)	No	77.8 (49)	58.2 (39)
	Yes	22.2 (14)	41.8 (28)

**Table 2:** Means, Standard deviations, and Ranges for the study variables by groups.

	Immigrant Latinos			U.S. Born Latinos		
	M	SD	Range	M	SD	Range
Substance Use Index (lifetime)	9.83	7.26	0.50 – 29.13	8.57	5.34	0.13 – 23.13
Anxiety	8.55	5.24	0 – 17	8.58	5.46	0 – 22
Affiliation with U.S. Culture	2.86	0.73	1.33 – 4	3.73	0.35	2.83 – 4
Contact with Important People	34.93	22.48	0 – 90	46.43	27.10	0 – 90
Full-Time Employment	4.17	4.93	0 – 25	3.68	4.51	0 – 22
Years of Formal Education	10.46	2.89	0 – 16	11.76	1.79	8 – 16
Age	38.89	10.99	18 – 63	33.84	9.35	18 – 51

**Table 3:** Chi-square differences between the trimmed model and model with paths constrained to equal.

Paths	Freely Estimated		Constrained				
	X^2^	df	X^2^	df	ΔX^2^	Δdf	p
Model with paths freely estimated	6.61	3	-	-	-	-	-
Trimmed Model	8.12	7	-	-	-	-	-
Model with paths constrained to equal	-	-	18.72	10	10.6	3	0.01

**Table 4:** Correlations for the immigrant and U.S. born groups.

Measure	1	2	3	4	5	6	7	8
Substance Use Index (lifetime)	-	0.24	−0.22	−0.25*	0.21	−0.07	0.46**	0.16
Anxiety^1^	0.26	-	−0.12	0.18	0.04	0.02	0.09	−0.20
Affiliation with U.S. Culture	0.20	−0.08	-	0.14	0.09	−0.15	−0.39**	−0.20
Contact with Important People^2^	0.10	0.13	−0.02	-	0.08	−0.01	−0.27*	0.26*
Full-Time Employment^3^ (years)	0.18	−0.27	−0.02	0.01	-	0.22	0.44**	0.12
Education (years completed)	0.04	−0.33*	0.23	0.18	0.20	-	0.18	0.14
Age	0.48**	0.25	−0.11	0.06	0.34**	0.20	-	0.23
Gender	−0.04	0.01	−0.28*	−0.20	0.06	−0.03	0.15	-

Note: Correlations for non-US born group (n = 62) are below the diagonal; correlations for the US born group (n = 68) are above the diagonal.

1 The Anxiety subscale from the TCS-40 measures anxiety symptomatology within the last month.

2 Average contact with Important people within 90 days0.

3 Full-time employment within the past 3 years.

** p < 0.01

* p < 05.0
